# Don't blame the BAME: Ethnic and structural inequalities in susceptibilities to COVID‐19

**DOI:** 10.1002/ajhb.23478

**Published:** 2020-07-16

**Authors:** Gillian R Bentley

**Affiliations:** ^1^ Department of Anthropology Durham University Durham UK

One of the striking features emerging from the COVID‐19 pandemic has been the early recognition of ethnic disparities in both vulnerability to contracting the disease, as well as its outcome. These disparities were first reported in news outlets (Blow, 2020; Cowan, 2020; Godin, 2020; The Guardian, 2020a) and, more recently, verified in academic journals (e.g., Bhala, Curry, Martineau, Agyeman, & Bhopal, 2020; Khunti, Singh, Pareek, & Hanif, 2020; Laurencin & McClinton, 2020; Webb Hooper, Nápoles, & Pérez‐Stable, 2020), as well as through organizations responsible for gathering health statistics (eg, APM Research Lab, 2020; CDC, 2020; ICNARC, 2020; ONS, 2020). In particular, Black, Asian, and minority ethnic (BAME) groups have emerged as more susceptible to higher morbidity and mortality than either UK or USA white groups. The Office for National Statistics (ONS)—the central organization responsible for producing demographic statistics in the UK—recently stated that Blacks were over four times more likely than whites in England and Wales to die from COVID‐19, figures that were equivalent across both genders. The ONS statistics included both confirmed and suspected cases of COVID‐19, as well as deaths in hospitals and the community. Similarly, in the USA, the Centers for Disease Control (CDC) has reported that almost twice as many Black and Hispanic individuals were hospitalized with COVID‐19 than are proportionally represented in the community (CDC, 2020).

From a biocultural perspective, it has been heartening to witness an early recognition in both the UK and the USA that inter‐population variation in susceptibilities to corona virus lies not in biology or our genes, but mostly in social and structural differences between human groups that have often led to health disparities (Bhala et al., 2020; Webb Hooper et al., 2020) (see Figure 1). The existence of such disparities is commonly referred to in anthropological and related literatures as “structural violence” (Farmer, Nizeye, Stulac, & Keshavjee, 2006; Galtung, 1969). Such considerations of structural inequalities in health have, for the most part, dominated emerging reports rather than questions of genes, ethnicity or(particularly, in North American parlance) the contested word “race” (Fuentes et al., 2019). For example, when socioeconomic factors as well as preexisting health conditions were controlled for in the ONS (2020) study cited above, the mortality risk for Blacks was reduced, but remained almost twice as high as whites, and higher for males (raising questions of course about what explains the residual differences); the figures for South Asians were similar (ONS, 2020). The ONS, at the time, was not able to include the types of jobs held by individuals among the confounders, which they argue could also affect their models.

**FIGURE 1:**
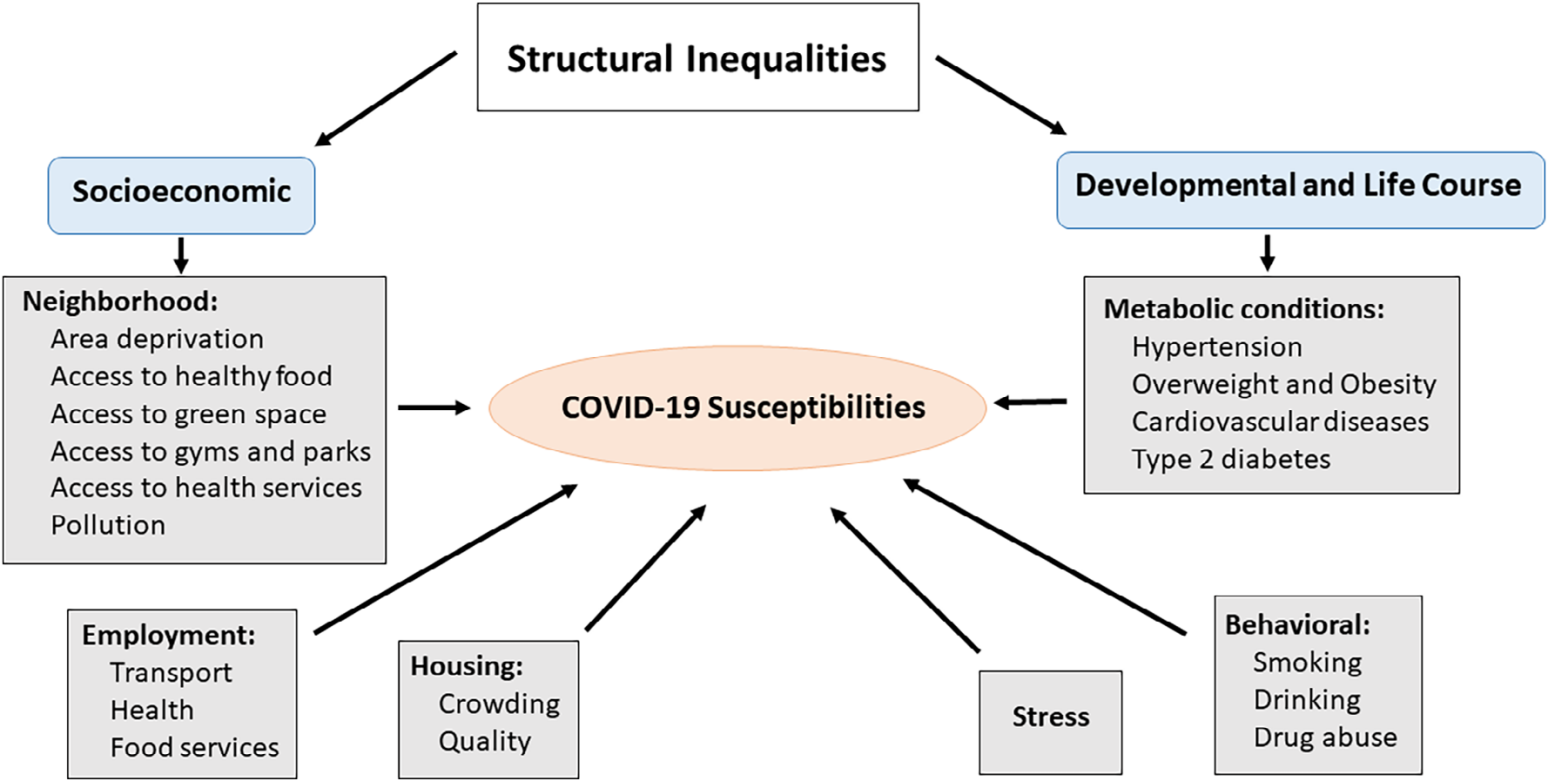
The relationship of structural inequalities to susceptibility to COVID‐19

However, more recently, insidious and potentially racist allusions are beginning to emerge appearing to blame African Americans as somehow responsible for the relatively large number of cases and deaths from COVID‐19 in the USA, stoking age‐old tropes, and attributing morbidity and mortality to the behaviors and predispositions of BAME groups (Guardian, 2020b; Strings, 2020). The Guardian (2020b) has speculated about the political motivations behind these attributions.

In reality, structural or social inequalities that affect individual vulnerabilities to SARS‐CoV‐2 include exposures through types of employment, whether people are working in essential transport networks carrying large numbers of people, or in small grocery shops that place BAME communities at greater risk of contracting COVID‐19 (Figure 1). These communities are also frequently at higher risk for metabolic disorders including obesity, cardiovascular diseases, hypertension and type 2 diabetes, some of which are linked to higher risks for contracting COVID‐19, and poorer outcomes once contracted (Azkur et al., 2020). Given that mortality appears to result more from the inflammatory response engendered from exposure to COVID‐19, as well as its ability to attack many organs in the body, metabolic diseases render many individuals at high risk for adverse outcomes once they become ill (Matricardi, Dal Negro, & Nisini, 2020). Front‐line health workers are also at high risk for contracting COVID‐19. In England, the National Health Service (NHS) has long imported qualified doctors, nurses, and carers from abroad (now 13% of all staff), and proportionally twice as many BAME individuals work for the NHS as in the rest of the economy (Baker, 2019). A recent report also suggested that BAME health workers have had less access to personal protective equipment than their white European counterparts (Royal College of Nursing, 2020). In the USA, there are additional social problems faced by minority groups, such as the potential lack of health care due to costs of health insurance. This is a problem that BAME groups do not face in the UK with its national health service. In addition, BAME communities often live in neighborhoods classified as food deserts which affect access to healthy foods, or they lack green spaces for exercise and fresh air. Ethnic minorities frequently live in areas or regions that are more polluted than others, situations which can be exacerbated by lack of service provision including garbage collection.

These kinds of “structural violence” are common for ethnic minorities in many northern nations, and can mostly explain inter‐group susceptibility to COVID‐19. Nevertheless, are there potentially any underlying biological realities that could also render some groups more vulnerable to poorer outcomes after contracting the disease? There are clearly differences in susceptibility based on both age and gender that remain little understood, at present, and that are not wholly due to either social or behavioral differences. In addition, there may be variation in the way that some individuals respond to drugs—so‐called pharmacogenetic differences—that might explain recovery times or reactions to particular pharmaceutical interventions (Shah, 2005). Just as some individuals appear immune to HIV‐AIDS (Carrington et al., 1996), similarly some individuals may also, for unknown reasons, be immune to SARS‐CoV‐2, or are asymptomatic carriers (Azkur et al., 2020).

There have been a few attempts to explore these underlying biological issues in relation to SARS viruses. One example is the study of population differences in genetic polymorphisms in the angiotensin‐converting enzyme‐2 (ACE2) receptor, found in many human organs such as the lung, brain, kidney, heart and gut, and through which related corona viruses (SARS‐CoV‐1, and the more recent SARS‐CoV‐2) binds to the new host, primarily through lung entry. Although East Asians appear to have higher expression of ACE2 levels compared to people of European descent, there does not seem to be any association between genetic variants and COVID‐19 outcome (Cao et al., 2020). Similarly, Nguyen et al. (2020) performed an in silico analysis of different classes of human leukocyte antigen (HLA) genotypes for their response to exposure to SARS‐CoV‐2, and also mapped different HLA variants globally to estimate the potential epidemiological impact at the population level. Again, while one HLA variant appeared to confer greater protection, another might render an individual more susceptible to COVID‐19, but neither were suggested to have an impact at a global level.

More recently, Genomics England (2020) has announced that they will begin a study of 20 000 hospitalized patients who have experienced the worst symptoms of COVID‐19, and will compare them to 15 000 patients who have had only mild symptoms, in an effort to explore potential genomic differences in susceptibility to the disease. The study will undoubtedly take some time to complete, and it is not known how the research will either collect or address other risk factors alongside genetic variability. Similarly, the Biobank longitudinal study in the UK has requested its current participants to enroll in a subsidiary study to examine those factors (genetic or otherwise) that could influence susceptibility to COVID‐19 (Biobank, 2020), although the proportion of BAME individuals participating in Biobank is lower than in the national population (Fry et al., 2017).Other physiological reasons for differential ethnic susceptibility to COVID‐19 have been suggested, including the potential of Vitamin D deficiency to increase susceptibility which would adversely affect people with darker skin living in countries at higher latitudes; this connection has since been downplayed (Hamiel, Kozer, & Youngster, 2020).

We should also remember that developmental and epigenetic influences mediated through the environment (socioeconomic and otherwise) can also impact individuals in later life and, through this route, affect their susceptibility to pathogens (Conching & Thayer, 2019; Nelson, 2009; Wells, 2016). South Asians, for example, are suggested to have a particular thin‐fat phenotype in response to long‐standing conditions of fetal under‐nutrition that render them particularly vulnerable to metabolic disorders in later life (Yajnik et al., 2003. These phenotypic traits also appear to be inter‐generationally preserved (van Steijn et al., 2009) and, as mentioned above, are currently linked to risks for COVID‐19 morbidity and mortality (Azkur et al., 2020).

Life course events such as allostatic load also seem to affect the rate at which individuals age biologically, such that their biological age is higher or lower than their chronological age. These discrepancies have been measured using a variety of techniques such as analyses of telomerelength, or epigenetic age, and many ethnic minorities appear to have higher biological ages relative to their chronological ages (eg, Levine & Crimmins, 2014; Simons et al., 2016). Given that COVID‐19 vulnerability is positively correlated with age, those individuals with a higher biological age might also be increasingly susceptible to the effects of COVID‐19.

More broadly, at a wider geographical level, long‐standing differences between countries in terms of Gross Domestic Product, infra‐structure and histories of colonial oppression, are thought to make many southern hemisphere, lower and middle‐income countries (LMICs) much more vulnerable to the emerging spread of corona virus and its health consequences (Bhutta, Basnya, Saha, & Laxminarayan, 2020; Roberton et al., 2020). There has also been speculation about the potentially catastrophic consequences of poor health, crowded conditions, lack of sufficient health personnel, and inability to socially distance in most of the camps housing refugees in various parts of the world (Kassem, 2020; Nott, 2020).

The statistics, in fact, looked encouraging early on, in that the numbers of cases and deaths appeared much lower in LMICs than in Europe, although the African Continent has been temporally behind Europe in exposure to SARS‐CoV‐2 (Hashim, 2020). In South Asia, the lower mortality rate was variably assigned to the demographic structure of the population, with a majority of younger individuals who may have been less likely to transmit SARS‐CoV‐2 (Hashim, 2020). In addition, it was speculated that exposure to more diseases during early life, or a more intense pattern of childhood immunizations, might somehow prime individuals to be more resistant to the virus. Early reports, however, that there might be some protection afforded by theBacillus Calmette‐Guérin(BCG) vaccination against tuberculosis (which was also routinely given to most children in the UK until 2005) have since been questioned (Kumar & Meena, 2020; Redelman‐Sidi, 2020). Another factor that might shield refugee camps from the pandemic is that they remain relatively isolated from tourist and visitor trails, while travel by inmates outside the camps is often restricted (Abdalfatah, 2020). However, more recently, the pandemic has been accelerating in southern countries, with rapidly increasing rates in the African Continent and in South Asia, providing challenges to the health care systems in many countries (Johns Hopkins University, 2020; Vaidyanathan, 2020), and an ominous portent of what might be the ultimate morbidity and mortality rates in LMICs.

Finally, and by no means least in relation to structural inequalities, as we develop new potential treatments and vaccines to treat COVID‐19, we must also be aware of the need to test and gather data on ethnic minorities, who are generally poorly represented in clinical trials and longitudinal health cohort studies (Jackson & Kuhlman, 2019).

The study of health in relation to structural inequalities, racism, and so‐called “racial medicine” has often been the focus of anthropology (eg, Conching & Thayer, 2019; Ifekwunigwe et al., 2017; Weigmann, 2006).We should be reassured, barring selective political rhetoric that has the potential to be extremely damaging, by the evident recognition in press outlets, popular science sources, and elsewhere, that ethnic inequalities in health do not reflect underlying human biology or genes, but rather the social environment in which human individuals find themselves embedded. We can only hope that the current pandemic might be an eventual catalyst to addressing and remediating these inequalities after decades of increasingly widening inequality gaps within human societies (Dorling, 2013; Marmot, 2015).

## AUTHOR CONTRIBUTIONS

**Gillian Bentley:** Conceptualization; writing‐original draft; writing‐review and editing.

## DISCLOSURE OF INTEREST

The author declares no conflict of interest.
